# Differential replicative ability of clinical dengue virus isolates in an immunocompetent C57BL/6 mouse model

**DOI:** 10.1186/s12866-015-0520-7

**Published:** 2015-09-29

**Authors:** Veridiana Ester Barros, Nilton Nascimento dos Santos-Junior, Alberto Anastacio Amarilla, Adriana Moreira Soares, Rafael Lourencini, Amanda Cristina Trabuco, Victor Hugo Aquino

**Affiliations:** Laboratory of Virology, Department of Clinical Analyses, Toxicology and Food Sciences, School of Pharmaceutical Sciences of Ribeirao Preto, University of Sao Paulo, Av do Café, s/n, CEP: 14040-903 Ribeirao Preto, Sao Paulo Brazil

**Keywords:** *Dengue virus*, Animal model, Antibody dependent enhancement, Clinical isolates

## Abstract

**Background:**

Several experimental animal models have been used to study the pathogenesis of dengue disease; however, most of the studies used laboratory-adapted viruses, which lack the virulence of viruses circulating in humans. The aim of this study was to analyze the ability of clinical *Dengue virus* (DENV) isolates (D2/BR/RP/RMB/09 and D3/BR/SL3/02) to infect immunocompetent C57BL/6 mice.

**Methods:**

Two strategies of intraperitoneal infection, which were based on the concept of the antibody dependent enhancement phenomenon, were used. In one strategy, the animals were inoculated with macrophages infected in vitro with dengue viruses, which were incubated with enhancing antibodies, and in the other strategy, the animals were inoculated with a complex of enhancing antibodies and dengue viruses.

**Results:**

The D3/BR/SL3/08 isolate showed a higher ability of infection (virus RNA was more frequently detected in the serum and in several organs) in the experimental model compared to both the D2/BR/RP/RMB/2009 isolate and a laboratory adapted DENV-1 strain (Mochizuki strain), regardless of the infection strategy used. The main features of the D3/BR/SL3/08 isolate were its neuroinvasiveness and the induction of an extended period of viremia. Enhancing antibodies did not influence on the infection of animals when macrophages were used, but the level of viremia was increased when they were used as a complex with a D3/BR/SL3/02 isolate.

**Discussion:**

We showed that DENV isolates could infect immunocompetent C57BL/6 mice, which have has been previously used to study some aspect of dengue disease when infected with laboratory adapted strains. DENV genome was detected in the same organs found in humans when autopsy and biopsy samples were analyzed, showing that C57BL/6 mice reproduce some aspects of the DENV tropism observed in humans. The main difference observed between the D3/BR/SL3/02 and D2/BR/RP/RMB/2009 clinical isolates was the neuroinvasive ability of the first one. Neuroinvasiveness has been described in some DENV infected cases and is common for other members of the *Flavivirus* genus.

**Conclusions:**

These results suggest that C57BL/6 mice can be used as an experimental model to evaluate virulence differences among DENV clinical isolates.

## Background

Dengue is the most important illness caused by arboviruses (arthropod born viruses) in humans. Its incidence has increased dramatically around the world in recent decades. Over 2.5 billion people, more than 40 % of the world’s population, are now at risk of dengue infection. The World Health Organization (WHO) currently estimates there may be 50–100 million dengue infections worldwide every year [1]. The *Aedes aegypti* mosquito is the primary vector of dengue; however, other mosquitoes, such as the *Aedes albopictus* and *Aedes africanus*, are also important vectors in Asia and Africa, respectively. *Dengue virus* (DENV), a member of the *Flavivirus* genus and the *Flaviviridae* family, has a positive-sense, single-stranded RNA genome of approximately 11 kilobases that is covered by an icosahedral capsid and a lipid envelope [2]. Serological studies have classified the virus into four immunological related subtypes: DENV-1, DENV-2, DENV-3 and DENV-4 [3–5]. WHO expert consensus groups have agreed that “dengue is one disease entity with different clinical presentations and often with unpredictable clinical evolution and outcome” [6]. Therefore, to facilitate the classification of dengue cases, in 2009 the WHO proposed a classification of dengue into levels of severity, dengue (with or without warning signs) and severe dengue, in place of the former dengue fever (DF) and dengue hemorrhagic fever (DHF) classification [6]. The main symptoms of dengue include fever, retro-orbital pain, headache, skin rash and bone and muscle pain; the more severe form is characterized by severe plasma leakage, severe hemorrhage and/or severe organ impairment. Most patients recover following a self-limiting, non-severe clinical course; however, a small proportion progress to severe disease, mostly characterized by plasma leakage. The pathogenesis of severe disease remains unclear, and several factors appear to be involved in the development of hemorrhagic manifestations and vascular leak syndrome development. Epidemiological studies have shown that a secondary infection with a different virus subtype is highly associated with the severe form of the disease [7]. However, few individuals develop the more severe forms after a secondary infection in endemic regions. It is believed that host, environment and virus factors are involved in the outcome of the disease. Several experimental animal models have been used to study the pathogenesis of the disease [8]; however, most studies used laboratory adapted viruses, which lack the virulence of viruses that circulate in humans. In this study, we demonstrated a differential ability of infection of clinical DENV isolates in C57BL/6 mice, suggesting that this experimental model can be used to study virulence differences among clinical isolates.

## Methods

### Viruses

A laboratory-adapted DENV-1 (Mochizuki strain) and clinical DENV-2 (D2/BR/RP/RMB/2009 isolate) [9] and DENV-3 (D3/BR/SL3/02 isolate) isolates [10] were used in this study. The viruses were propagated in C6/36 cells, which were cultured in a flask containing Leibovitz’s L-15 medium (Vitrocell, Campinas, Brazil) supplemented with 2 % fetal bovine serum (FBS) (Gibco-BRL Life Technologies, Grand Island, NY) and maintained at 28 °C for up to seven days. The D2/BR/RP/RMB/2009 and D3/BR/SL3/02 clinical isolates were passed in C6/36 cells culture three and five times, respectively, to increase the viral titers. The supernatant was aliquoted and stored at −70 °C until use. Viral titers were determined with a plaque assay [11] and with a quantitative real-time RT-PCR using a viral RNA transcribed in vitro to construct an standard curve as described previously [12].

### Ethics statement

Three-to-four-week-old immunocompetent C57BL/6 mice and Swiss mice were obtained from the Central Animal Facility at the University of Sao Paulo, Ribeirao Preto branch. All animal experiments were performed according to the guidelines of the Brazilian College of Animal Experimentation and approved by the Ethical Committee on Animal Experimentation at the Medical School of Ribeirao Preto, University of Sao Paulo (CETEA/FMRP/123/2010).

### Preparation of mice immune sera

Mice immune sera were prepared from DENV-1 (Mochizuki strain)- and DENV-2- (D2/BR/RP/RMB/2009 isolate)-infected Swiss mice using standard protocols [13]. Briefly, the animals were intraperitoneally inoculated with 2.0x10^5^ plaque forming units (PFU) (~2.0x10^8^ RNA copies/mL) of virus and Freund’s complete adjuvant. Three other viral inoculations were performed weekly using Freund’s incomplete adjuvant. One week after the last virus inoculation, the animals were anesthetized using a mixture of ketamine-xylazine for blood collection [14]. The immune serum was separated after blood clotting by centrifugation and was stored at −80 °C until use.

### Peritoneal macrophages preparation and infection

C57BL/6 mice were intraperitoneally injected with 1 mL of phosphate buffer saline (PBS) containing 3 % thioglycolate (Sigma-Aldrich, St Louis, MO, USA). Four days later, mice peritoneum was rinsed with cold Leibovitz’s L-15 medium to collect the peritoneal cells. Cells from different animals were centrifuged for 10 min at 1000 g and then washed three times with PBS. These cells were resuspended in Leibovitz’s L-15 medium supplemented with 10 % FSB and were added to 12-well culture plates (TPP, Swiss) at a density of 2.0x10^6^ cells per well. Macrophages were allowed to adhere to the plate surface for two hours at 37 °C, and then the non-adherent cells were washed off with PBS. Leibovitz’s L-15 medium supplemented with 10 % FBS was added to the macrophage cells, which were further incubated at 37 °C for 24 h. Macrophages were infected with a 0.1 multiplicity of infection (MOI) with the Mochizuki strain, the D2/BR/RP/RMB/2009 isolate or the D3/BR/SL3/02 isolate and were incubated at 37 °C for two days. Infection was confirmed by detection of viral RNA in the cell culture supernatant with a quantitative real-time RT-PCR, using a pair of primers ( 5′UTR-S: AGT TGT TAG TCT ACG TGG ACC GA and 5′UTR-C: CGC GTT TCA GC A TAT TGA AAG) to amplify a region of 120 base pairs of the 5′ end of the viral RNA [12, 15].

### Antibody-dependent enhancement (ADE) assay

The D3/BR/SL3/02 isolate (2.0x10^5^ PFU, 2.0x10^9^ RNA copies) was incubated with several dilutions (1:10, 1:100, 1:1000, 1:2000, 1:5000, 1:10000, 1:100000) of DENV-1 immune serum, naive serum or PBS for 1 h at 37 °C. The immune complexes were used to infect the macrophages contained in a 12-well plate, which was prepared as mentioned above. Three replicates of macrophages were infected with each immune complex. After 2 h, the cells were washed with PBS to remove unbound immune complexes. Leibovitz’s L-15 medium supplemented with 10 % FSB was added to the cells, which were further incubated at 37 °C for two days. The ADE enhancement of macrophage infection was determined based on virus titer quantification in the cell culture supernatant using a real-time RT-PCR [12]. ADE assays for the D2/BR/RP/RMB/2009 isolate and the Mochizuki strain were performed using DENV-1 and DENV-2 immune sera, respectively.

### Animal infections

C57BL/6 mice were infected using two strategies. In one strategy, the animals were infected by intraperitoneal (i.p.) inoculation of 2.0x10^5^ PFU of viruses (200 μL) that were previously incubated for one hour with enhancing antibodies or PBS. In the other strategy, the animals underwent i.p inoculation with macrophages that were infected *in vitro* with viruses that were previously incubated for one hour with enhancing antibodies or PBS. Animals inoculated with PBS and uninfected-macrophages (sham-infected) were used as controls.

### Blood and organ collection

Animals were anesthetized using a mixture of ketamine-xylazine [14] at different times post-infection (p.i.) (*n =* 5 at each time point). Blood was obtained from the retroorbital region and collected in a tube containing sodium citrate (3.8 %) as an anticoagulant. An aliquot of the blood was collected in a tube without anticoagulant for viral load quantification in the serum. Then, the animals underwent intracardiac perfusion with 15–20 mL of a 0.9 % NaCl solution, followed by the removal of the liver, brain, spleen and kidney. The whole organs were placed in 2 mL tubes containing 500 μL of PBS and were homogenized with a tissue homogenizer (Ultra Stirrer, Biosystems, PR, Brazil). The organ suspensions were centrifuged at 8000 g for 5 min, and the supernatants were used for viral load quantification.

#### Statistical analysis

Data are expressed as the means ± standard deviations (SDs). Statistical significance was assessed by using one-way ANOVA, followed by a Tukey multiple comparison test. A *p-*value of less than 0.05 was considered significant.

## Results

### DENV infect C57BL/6 peritoneal macrophages

**Fig. 1:**
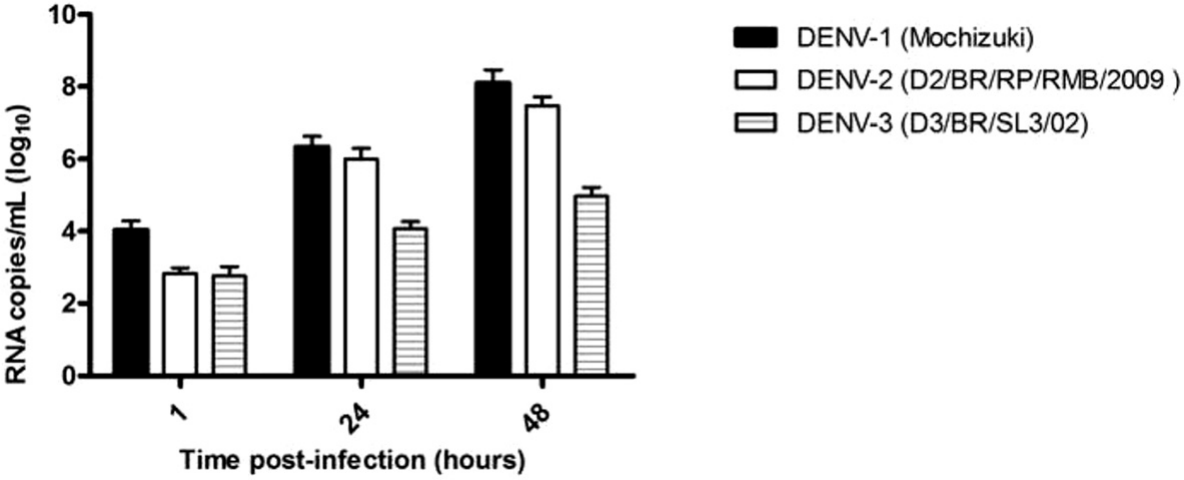
Susceptibility of C57BL/6 mice peritoneal macrophages to infection with DENV. Real-time RT-PCR for the detection and quantification of the virus genome in the supernatant of the culture of C57BL/6 mice macrophages after 1, 24 and 48 h of infection with the Mochizuki strain, the D3/BR/SL3/02 and the D2/BR/RP/RMB/2009 isolates

To determine the susceptibility of C57BL/6 macrophages to infection by DENV, peritoneal macrophages were incubated *in vitro* with laboratory-adapted (DENV-1, Mochizuki strain) and clinical DENV isolates (DENV-2, D2/BR/RP/RMB/2009 and DENV-3, D3/BR/SL3/02). Cell infection was confirmed by detection of viral RNA genome in the cell culture supernatants with a real-time RT-PCR at 1, 24 and 48 h post inoculation (Fig. 1). The highest viral load in the cell culture supernatants was observed at 48 h post inoculation.

### Antibodies enhance infection of C57BL/6 peritoneal macrophages

**Fig. 2:**
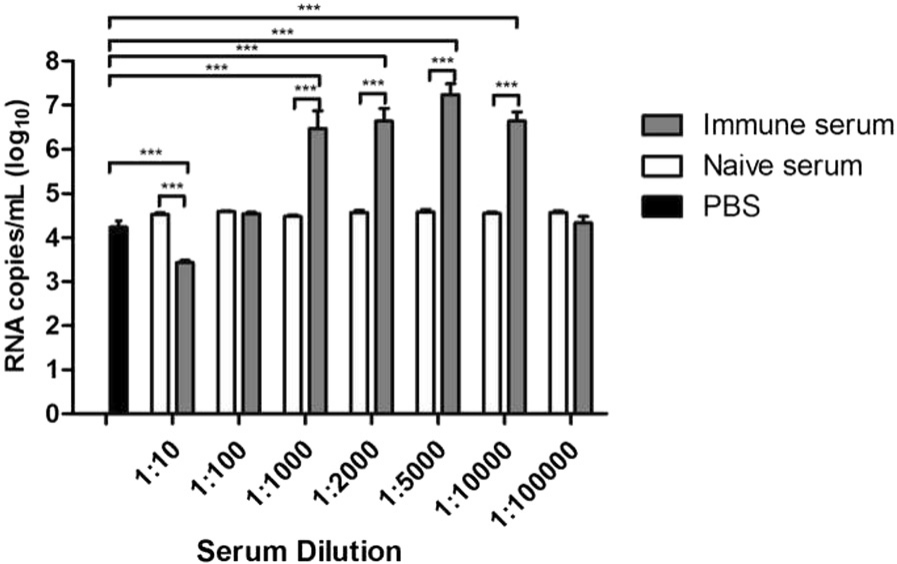
Antibody-dependent enhancement (ADE) of infection of C57BL/6 mice macrophages with DENV-3. The enhancement of infection was evaluated by quantification of the viral titer in the supernatant of the macrophage cultures 48 h post-infection with the D3/BR/SL3/02 isolate, which was previously incubated with different dilutions of DENV-1 immune serum (1:10, 1:100, 1:1000, 1:2000, 1:5000, 1:10000 and 1:100000). Data represent the mean values ± SD. The results are representative of two similar and independent experiments. ***p < 0.001 when the virus incubated with DENV-1 immune serum was compared to the virus incubated with PBS or naive serum

**Fig. 3:**
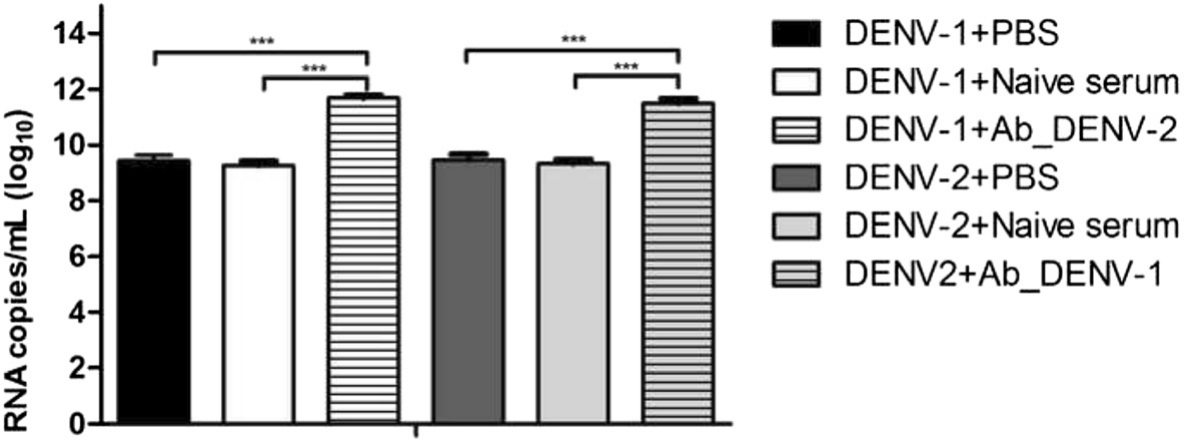
Antibody-dependent enhancement (ADE) of infection of C57BL/6 mice macrophages with DENV-1 and DENV-2. The enhancement of infection was evaluated by the quantification of the viral titer in the supernatant of the macrophage cultures 48 h post-infection with the Mochizuki strain and the D2/BR/RP/RMB/2009 isolate, which were previously incubated with 1:5000 dilutions of DENV-2 and DENV-1 immune sera, respectively. Data represent the mean values ± SD. The results are representative of two similar and independent experiments. ***p < 0.01 when viruses incubated with heterologous immune sera were compared to viruses incubated with PBS

To investigate the effect of DENV-specific antibodies on macrophage infection, an ADE assay was performed. The D3/BR/SL3/02 isolate was incubated with several dilutions of DENV-1 immune serum to induce the formation of antibody/virus complexes. Then, these complexes were used to infect C57BL/6 peritoneal macrophages for 48 h. Figure 2 shows that several dilutions of the heterologous immune serum enhanced macrophage infection, confirming the presence of antibodies in enhancing concentrations in those dilutions. The highest viral titer in the cell culture supernatants was observed when the virus was incubated with a dilution of 1:5000 of DENV-1 immune serum. ADE tests with the Mochizuki strain and the D2/BR/RP/RMB/2009 isolate were performed using DENV-2 and DENV-1 mice immune sera, respectively. In these experiments, only the dilution (1:5000) that induced the highest enhancement of D3/BR/SL3/02 infection on macrophages was used. Figure 3 shows that heterologous antibodies also enhanced the infection of macrophages with the Mochizuki strain and the D2/BR/RP/RMB/2009 isolate.

### Clinical DENV isolates infect C57BL/6 mice

To investigate the susceptibility of C57BL/6 mice to infection with clinical DENV isolates, two strategies of intraperitoneal infection were used. In one strategy, the animals were inoculated with macrophages infected *in vitro* with viruses that were previously incubated with enhancing antibodies or PBS, and in the other strategy, the animals were inoculated with viruses that were previously incubated with enhancing antibodies or PBS.

### Inoculation of animals with macrophages infected with DENV

**Fig. 4:**
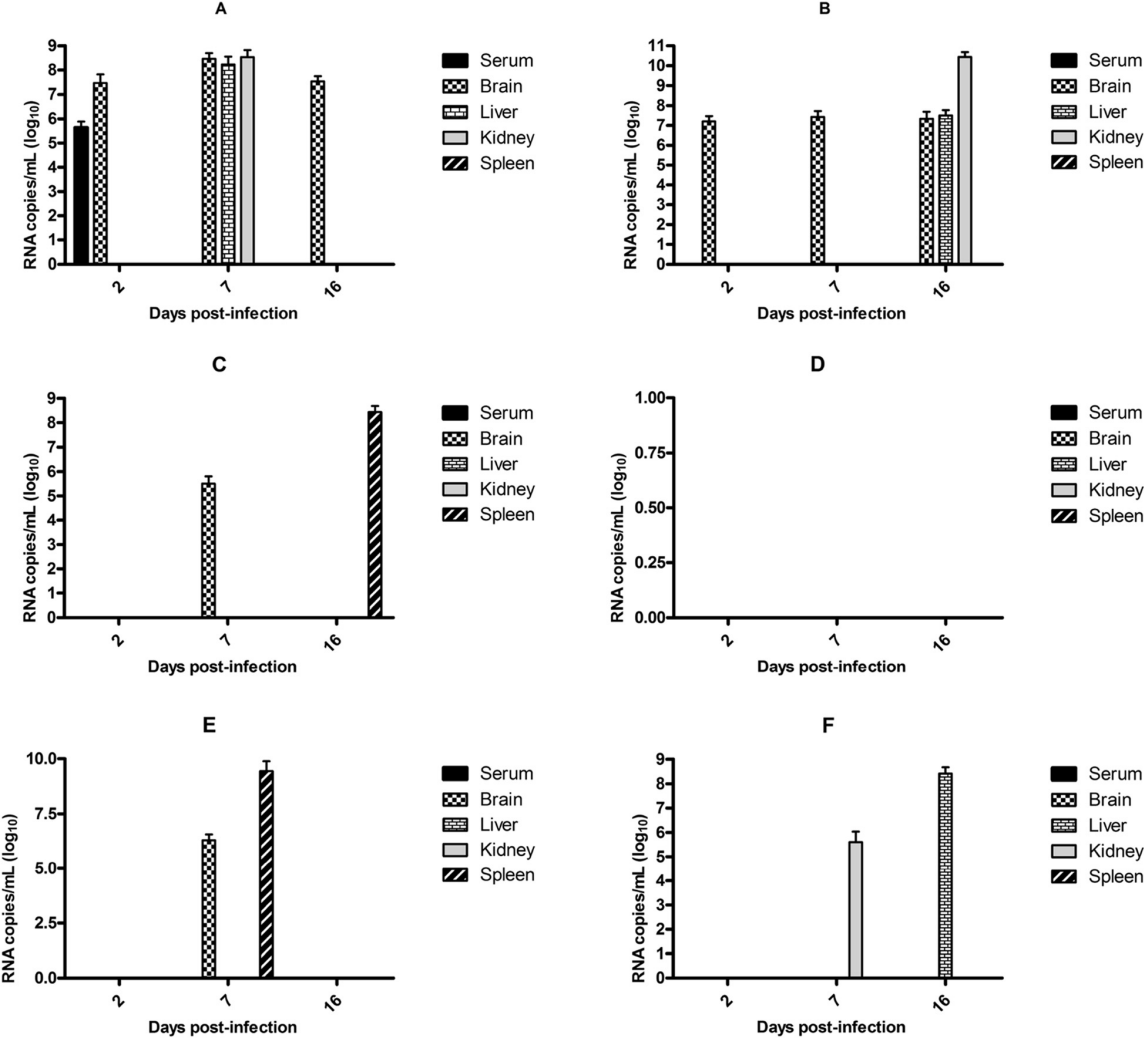
Susceptibility of C57BL/6 mice to infection with DENV. The animals were inoculated with macrophages infected *in vitro* with D2/BR/RP/RMB/2009 (*n =* 5) (a and b) and the D3/BR/SL3/02 (*n =* 5) (c and d) isolates, and Mochizuki strain (*n =* 5) (E and F), which were previously incubated with heterologous antibodies (a, c and e) or PBS (b, d and f). The infection was confirmed by detection of the virus genome by real-time RT-PCR in serum and organs. Data represent the mean values ± SD. The results are representative of two similar and independent experiments

The clinical D3/BR/SL3/02 and D2/BR/RP/RMB/2009 isolates, and the laboratory-adapted Mochizuki strain were incubated for 1 h with enhancing antibodies (1:5000) or PBS as mentioned in the ADE assay, and then used to infect the peritoneal macrophages *in vitro*. The infected cells were inoculated via the i.p. route into C57BL/6 mice, and infection of the animals was determined by virus genome detection with a real-time-RT-PCR. The animals were highly susceptible to infection with the D3/BR/SL3/02 isolate; which was detected in the blood and several organs (Fig. 4a, and b). Detection of the virus genome in the brain on the three analyzed days was the most substantial finding in animals inoculated with macrophages infected with the D3/BR/SL3/02 isolate, regardless of the use of enhancing antibodies or not (Fig. 4a, and b). The spleen was the only organ in which the D3/BR/SL3/02 genome was not detected.

Mice were less susceptible to infection with the D2/BR/RP/RMB/2009 isolate and the Mochizuki strain; the virus genome was detected only in the brain and the spleen, 7 and 16 days p.i., respectively (Fig. 4c), in the animals inoculated with macrophages infected with the D2/BR/RP/RMB/2009 isolate with enhancing antibodies; in the brain and the spleen, 7 days p.i in the animals inoculated with macrophages infected with the Mochizuki strain with enhancing antibodies (Fig. 4e); and in the kidney and liver, 7 and 16 days p.i, respectively, in the animals inoculated with macrophages infected with the Mochizuki strain without enhancing antibodies (Fig. 4f).

### Inoculation of animals with a complex DENV/enhancing antibodies

**Fig. 5:**
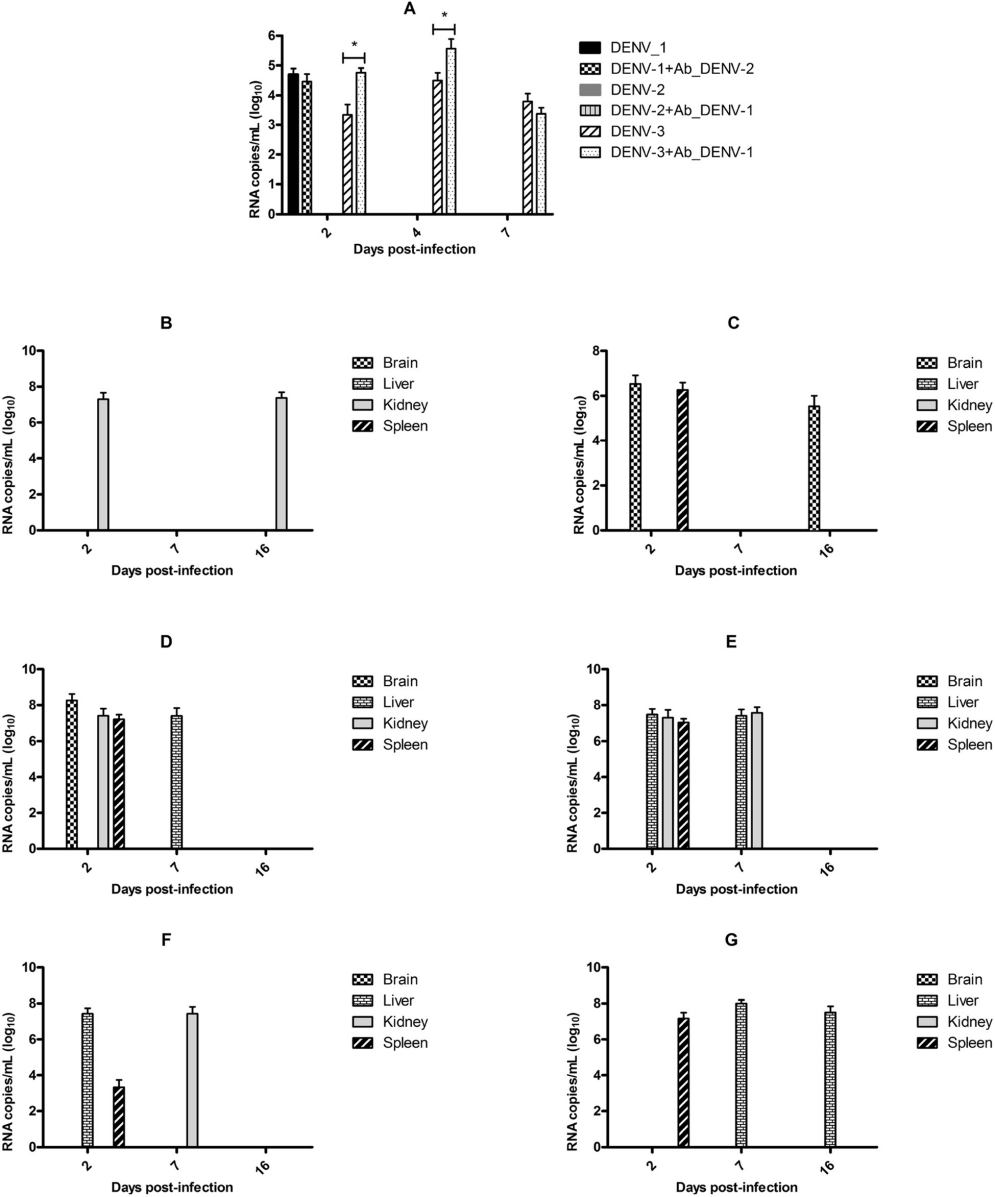
Susceptibility of C57BL/6 mice to infection with DENV. The animals were infected with D3/BR/SL3/02 (*n =* 5) (a, b and c) and D2/BR/RP/RMB/2009 (*n =* 5) (a, d and e) isolates, and Mochizuki strain (*n =* 5) (a, f and g). The infection was confirmed by detection of the virus genome by real-time RT-PCR in serum (a) and different organs (b-g). The animals were infected with D3/BR/SL3/02 and D2/BR/RP/RMB/2009 (*n =* 5) isolates, and Mochizuki strain, which were incubated with PBS (b, d and f, respectively) or enhancing antibodies (c, e and g, respectively). Data represent the mean values ± SD. The results are representative of two similar and independent experiments. *p < 0.05 when viruses incubated with heterologous immune sera were compared to viruses incubated with PBS

The D3/BR/SL3/02 and D2/BR/RP/RMB/2009 isolates, and the Mochizuki strain were incubated with enhancing antibodies or PBS as mentioned in the ADE assay, and then used to infect C57BL/6 mice via the i.p. route. The susceptibility of the C57BL/6 mice to infection with DENV was again confirmed by detection of virus genomes in the serum and several organs (Fig. 5). Animals infected with the D3/BR/SL3/02 isolate showed a longer period of viremia (up to 7 days p.i.) than the animals infected with the other viruses (Fig. 5a). In addition, enhancing antibodies induced a higher level of viremia (Fig. 5a) and the appearance of virus in the brain (Fig. 5c) of the D3/BR/SL3/08-infected animals. Mice infected with the Mochizuki strain showed viremia only on day 2 p.i., while mice infected with D2/BR/RP/RMB/2009 did not show viremia. The use of heterologous antibodies did not change greatly the profile of virus detection in bloods and organs of animals infected with D2/BR/RP/RMB/2009 isolate and Mochizuki strain.

## Discussion

The development of a suitable animal model for DENV infection has been hampered by the low level or lack of replication of DENV clinical isolates in wild-type mice and the lack of clinical disease in non-human primates [Review in [8]]. In this study, we showed that immunocompetent C57BL/6 mice were susceptible to infection with clinical *Dengue virus* isolates. This animal model has been previously shown to be useful for the study of hemorrhagic phenomena and endothelial damage after infection with very high doses of DENV-2 (3.0x10^9^ PFU of DENV-2 strain 16681) [16–18]. In addition, C57BL/6 mice were used to study cytokine and chemokine production after infection with a mouse-adapted DENV-2 (strain P23085) [18, 19]. We have also observed some aspects of human disease, such as thrombocytopenia, liver damage, and increases of IFN*γ* and TNF*α* cytokine production when this animal model was infected with a laboratory adapted DENV-1 (Mochizuki strain) [20].

Mosquitoes can intravascularly inoculate 10^4^ and 10^6^ PFU of *West Nile virus* per bite [21]; thus, it can be assumed that similar doses of DENV is inoculated by the *Aedes* species when feeding on humans. Therefore, inoculation of 10^4^ and 10^6^ PFU of DENV in animal models can mimic the dose of the virus inoculated with the mosquito bite. However, because immunocompetent mice have a natural resistance to DENV infection, high doses (10^8^-10^9^ PFU) of virus is usually required to induce clinical signs [16, 17, 22]. To overcome the requirement of high doses of the virus, which is difficult to produce in the laboratory, we sought to infect the animals using the antibody dependent enhancement (ADE) phenomenon. ADE of cell infection has been implicated in severe secondary dengue virus infection [23]. According to this hypothesis, antibodies from the primary infection at sub-neutralizing concentrations form infectious immune complexes with dengue virus of a secondary infection, enhancing the virus production in Fc receptor-bearing cells. Several authors have confirmed this phenomenon *in vitro* using either human or mouse cells [24–27]. Macrophages as well as monocytes and immature and mature dendritic cells are major targets of dengue virus infection [28, 29]. Those cells have also been found to be infected in experimental animals models [30]. In vitro infection of these cells with a complex of enhancing antibodies and virus results in the suppression of the innate response and an increase in DENV production [25, 26]. Therefore, based on the ADE concept, we used two strategies to facilitate infection of C57BL/6 mice with clinical *Dengue virus* isolates. In one strategy, we first infected macrophage cells *in vitro* with DENV incubated with enhancing antibodies and then used the cells to infect the animals. In the other strategy, we used a complex of enhancing antibodies and DENV to infect the animals. We found that C57BL/6 mice were susceptible to clinical *Dengue virus* type 3 and 2 (D3/BR/SL3/02 and D2/BR/RP/RMB/2009) isolates using both infection strategies, especially the first one, which was detected in the serum and in several organs like liver, spleen, kidney and brain. Similarly, the same organs were found to be infected with DENV in humans when autopsy and biopsy samples were analyzed [28, 29, 31–33], demonstrating that C57BL/6 mice reproduce some aspects of human DENV tropism. We have shown previously, analyzing the C57BL/6 mouse model that DENV-1 (Mochizuki strain) can be detected up to 10 and 16 days p.i. in the organs and serum, respectively [20]. Using the same animal model in this new study, we have found an extension of the period of virus detection (up to 16 days p.i.) in the organs, especially for D3/BR/SL3/02 isolate, which might be related to the strategy of infection used, enhancing antibodies and infected cells.

Due to the resistance of immunocompetent mouse models to DENV infection, intracranial inoculation, which does not represent a natural way of infection and induced neurological signs, is widely used for therapeutic and vaccine testing [8, 34]. Although the strategies of infections used in our study do not also represent natural infection, they can be an alternative to intracranial infection for therapeutic and vaccine testing, since DENV can be detected in the blood stream and several organs.

It is believed that laboratory adapted strains are more competent at infecting animals, which is why they are widely used in the literature to analyze several experimental models. However, in this study, we found a higher replicative ability of the clinical D3/BR/SL3/02 isolate than the laboratory-adapted Mochizuki strain in C57BL/6 mice; the virus genome was detected more frequently in the blood and in several organs in mice infected with the former virus. On the other hand, C57BL/6 mice showed low infection levels with a clinical DENV-2 (D2/BR/RP/RMB/2009) isolate, suggesting a differential replication ability of clinical *Dengue virus* isolates in this animal model. Consistent with these results, clinical DENV-3 isolates have also shown different replication ability and virulence patterns in C57BL/6 mice after intracranial inoculation [35]. Viruses that replicate more efficiently are often hypothesized to be more pathogenic in the host [36, 37]. In that sense, the severity of dengue disease has been associated with higher viral load in patient serum [38, 39]. In addition, the difference in the replicative ability of clinical DENV-3 isolates in dendritic cells has been associated with modulation of apoptosis and cytokine production [40]. Therefore, the replicative ability of DENV isolates found in this study, suggest that C57BL/6 mice can be used as an experimental model to evaluate virulence differences among clinical DENV isolates.

**Fig. 6:**
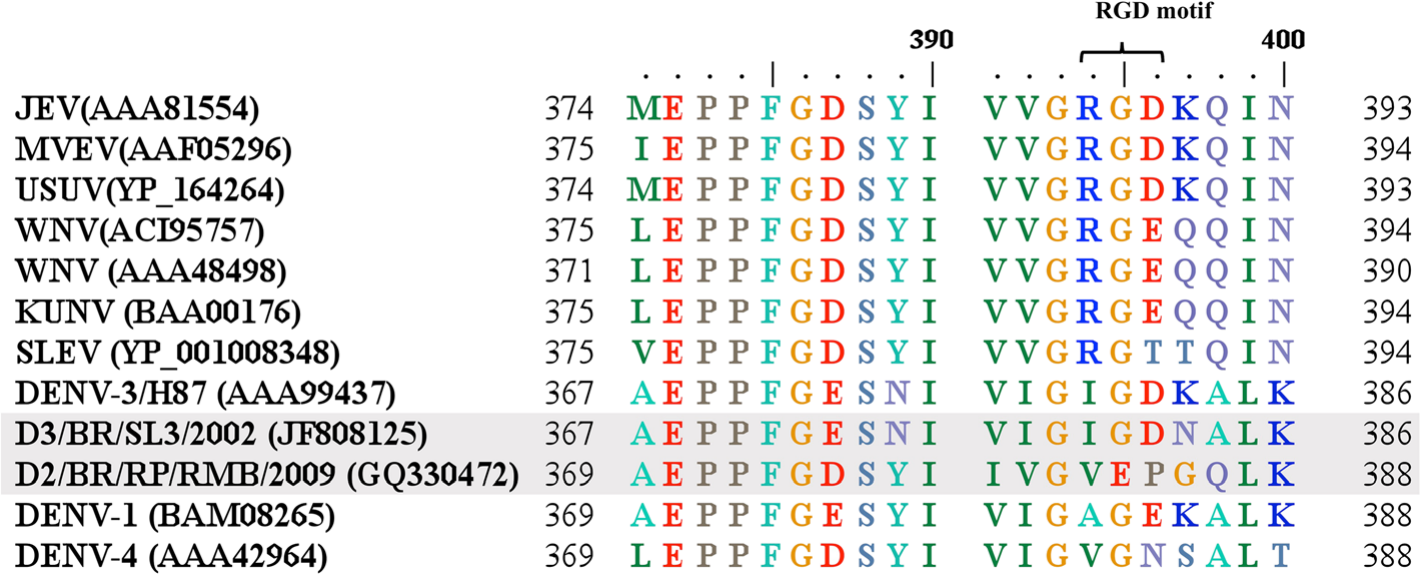
Comparison of the E protein amino acid sequence of flaviviruses. Alignment of a partial amino acid sequence of the E protein of flaviviruses, including the RGD motif. The E protein sequences of the following viruses were included in the alignment: *Japanese encephalitis virus* (JEV), *Murray Valley encephalitis virus* (MVEV), *Usutu virus* (USUV), *West Nile virus* (WNV), *Saint Louis encephalitis virus* (SLEV), and *Dengue virus* type 1, 3 and 4 (DENV-1, DENV-3, DENV-4). The clinical *Dengue virus* isolates and the RGD motif are shown in boxes. The GenBank accession number of the sequences is indicated in parenthesis

Several studies have shown that virulence is dependent on virus genetic characteristics [36, 41, 42]. The DENV-3 (D3/BR/SL3/08) used in this study was isolated in Sao Luis, Maranhao State, Brazil, in 2002, from a DF patient [10, 43]. DENV-3 was introduced in Brazil in 2000 and then spread throughout the country causing large epidemic outbreaks. This *Dengue virus* type 3 belongs to genotype III, which was associated with an increase in the number of severe cases worldwide [44]. DENV-2 (D2/BR/RP/RMB/2009) was isolated from a DF patient in Ribeirao Preto in 2009. DENV-2 was responsible for the more severe dengue epidemic in the city of Rio de Janeiro in 2007–2008 [45], which then spread to several regions of the country, reaching Ribeirao Preto in 2009, where it was less virulent. Ribeirao Preto faced the greatest epidemic in 2010–2011 with more than 29949 reported cases and 9 deaths, when DENV-1, DENV-2 and DENV-3 were circulating, but with a higher prevalence of DENV-1. Comparing the E protein amino acid sequence of these viruses with other flaviviruses, we found that the D3/BR/SL3/02 isolate but not the D2/BR/RP/RMB/2009 isolate has an RGD-like motif (IGD) (Fig. 6), the integrin-binding motif, which is important in cell-extracellular matrix and cell-cell adhesion [46]. It is speculated that this motif located in the putative receptor-binding site might be involved in the adsorption into host cells. Mutation of the E protein of the *Murray Valley encephalitis virus* has demonstrated the importance of this motif, with a fundamental role for residue D_390_, in tropism and virulence. Thus, the presence of an RGD-like motif might explain the higher infectivity of the D3/BR/SL3/02 isolate in this mouse model compared to the D2/BR/RP/RMB/2009 isolate.

In addition to the febrile illness and the more severe hemorrhagic form, 1 % to 5 % of infected patients develop neurological manifestations [47]. Encephalitis is the most common neurological manifestation, and the main symptoms are seizures, altered consciousness, and headaches [48]. Evidence of DENV replication in the brain has been observed in autopsy studies based on the detection of virus antigens/RNA and virus isolation from brain specimens [31, 32, 49, 50]. In addition, viral RNA was detected, and the virus was isolated from cerebrospinal fluid (CSF) samples of dengue cases with neurological manifestations [51]. The involvement of the central nervous system (CNS) in DENV infection is difficult to study because unmodified viruses do not infect or cause symptoms in experimental animal models. Intracranial inoculation of newborn or adult mice has been used to study neurovirulence; however, this might not have much biological relevance because the strains used are not necessarily neuroinvasive. In our study, viral RNA was detected more frequently in the brain when the animals were infected with the D3/BR/SL3/02 clinical isolate, suggesting that this is a neuroinvasive strain. The mechanism by which flaviviruses cross the blood–brain barrier to enter the brain parenchyma is not well understood. The use of experimental animal models have shown that *Japanese encephalitis virus* infects the CNS by typical endocytosis and transcytosis through the cerebral blood vessels and blood–brain barrier [52], whereas *West Nile virus* penetration into the CNS occurs by diffusion between capillary endothelial cells into the brain parenchyma induced by endogenous mediators [53, 54]. In both transcytosis and diffusion, a high viral load in the blood might play a crucial role in virus penetration into the CNS. In this study, we found viral RNA in the brain of mice infected with the D3/BR/SL3/02 isolate incubated with enhancing antibodies, which had higher viremia levels (Fig. 5), supporting the hypothesis of virus penetration into the CNS by transcytosis and/or diffusion. Another possibility is that viruses enter the CNS within infected leukocytes that may infiltrate the brain parenchyma. This mechanism is known as a “Trojan horse” entry because the pathogens are hidden within these immune defense cells, which are naturally able to traverse the blood–brain barrier [55, 56]. Infiltration of peripheral macrophages into the CNS has been demonstrated in experimental models and in fatal human cases after infection with *Japanese encephalitis virus* [57, 58]. In this study, viral RNA was detected in the brain of mice inoculated with macrophages infected with the D3/BR/SL3/02 isolate, suggesting that peripheral macrophages could be the carrier of the virus to the brain parenchyma. The low detection rate of viral RNA in the brain of mice inoculated with macrophages infected with either the D2/BR/RP/RMB/2009 isolate or the Mochizuki strain strongly suggests that detection of the D3/BR/SL3/02 isolate represents true replication of this virus in the brain and is not just the presence of the virus within the infiltrated macrophages.

Based on experimental evidence in the literature suggesting that a complex of enhancing antibodies and virus can lead to suppression of the innate response and an increase in DENV production in the infected Fc bearing cells [25, 26], we expected to find a more robust infection of animals inoculated with macrophages infected *in vitro* with viruses incubated with enhancing antibodies, which induced a higher virus titer *in vitro* (Fig. 3 and 4). However, a similar pattern of infection was observed in mice inoculated with infected macrophages regardless of the use of enhancing antibodies or not. These results suggest that DENV does not necessarily require enhancing antibodies to suppress the innate response of macrophages and that the virus can continue replication in those cells even after their inoculation into the mice. This is consistent with studies showing that DENV without enhancing antibodies inhibited the production of type I interferon, which is an important antiviral factor, in primary human dendritic cells [59]. On the other hand, a higher viremia level and the appearance of the virus in the brain were observed when the animals were infected with a complex of enhancing antibodies and the D3/BR/SL3/02 isolate, suggesting the enhanced infection of Fc receptor-bearing cells and an increase in viral production, as expected based on the ADE concept [60]. This result is consistent with the increased DENV infection observed in the AG129 mice after receiving subprotective levels of antibodies [61].

## Conclusions

Clinical DENV isolates have shown differential replication ability in immunocompetent C57BL/6 mice, suggesting that this experimental model can be used to study the virulence differences of clinical isolates. The virus was detected in the same organs found in humans, showing that C57BL/6 mice reproduce some aspects of the DENV tropism observed in humans. The main difference observed between the D3/BR/SL3/02 and D2/BR/RP/RMB/2009 clinical isolates was the neuroinvasive ability of the first one. Mice inoculated with macrophages infected with D3/BR/SL3/02 showed evidence of virus replication in the CNS, even in the absence of viremia, strongly suggesting that peripheral macrophages are the carrier of the virus into the brain parenchyma. However, D3/BR/SL3/02 RNA was also detected in the brain when enhancing antibodies induced a higher viral load in the blood, suggesting that the CNS may also be infected by transcytosis and/or diffusion of DENV. Further histopathological and cytokine production studies are being performed in our laboratory to better analyze the virulence differences of the DENV clinical isolates.

## Abbreviations

WHO: World Health Organization
DENV: *Dengue virus*
DF: Dengue fever
DHF: Dengue hemorrhagic fever
PBS: Phosphate buffer saline
FBS: Fetal bovine serum
MOI: Multiplicity of infection
PFU: Plaque forming units
i.p.: Intraperitoneal
p.i.: Post-infection
ADE: Antibody-dependent enhancement
SD: Standard deviation
JEV: *Japanese encephalitis virus*
MVEV: *Murray Valley encephalitis virus*
USUV: *Usutu virus*
WNV: *West Nile virus*
SLEV: *Saint Louis encephalitis virus*
CSF: Cerebrospinal fluid
CNS: Central nervous system

## References

[1] WHO: Dengue and severe dengue. In*.* Geneva: World Health Organization; 2014.

[2] Lindenbach B, Thiel H, Rice C, Knipe D, Howley P (2007). Flaviviridae: the viruses and their replication. Fields Virology.

[3] SABIN A (1950). The dengue group of viruses and its family relationships. Bacteriol Rev.

[4] SWEET B, SABIN A (1954). Properties and antigenic relationships of hemagglutinins associated with the dengue viruses. J Immunol.

[5] HAMMON WM, RUDNICK A, SATHER GE (1960). Viruses associated with epidemic hemorrhagic fevers of the Philippines and Thailand. Science.

[6] WHO: Dengue: guidelines for diagnosis, treatment, prevention and control. In. Geneva, World Health Organization; 2009. 23762963

[7] Guzman MG, Alvarez M, Halstead SB (2013). Secondary infection as a risk factor for dengue hemorrhagic fever/dengue shock syndrome: an historical perspective and role of antibody-dependent enhancement of infection. Arch Virol.

[8] Zompi S, Harris E (2012). Animal models of dengue virus infection. Viruses.

[9] Poloni TR, Oliveira AS, Alfonso HL, Galvao LR, Amarilla AA, Poloni DF, et al. Detection of dengue virus in saliva and urine by real time RT-PCR. Virol J. 2010;7. 10.1186/1743-422X-7-22 PMC2835670 20105295

[10] Aquino VH, Anatriello E, Goncalves PF, da Silva EV, Vasconcelos PFC, Vieira DS, Batista WC, Bobadilla ML, Vazquez C, Moran M (2006). Molecular epidemiology of dengue type 3 virus in Brazil and Paraguay, 2002–2004. Am J Tropical Med Hygiene.

[11] Muller VD, Russo RR, Cintra AC, Sartim MA, Alves-Paiva RM, Figueiredo LT, Sampaio SV, Aquino VH (2012). Crotoxin and phospholipases A_2_ from Crotalus durissus terrificus showed antiviral activity against dengue and yellow fever viruses. Toxicon.

[12] Muller VD, Soares RO, Dos Santos-Junior NN, Trabuco AC, Cintra AC, Figueiredo LT, Caliri A, Sampaio SV, Aquino VH (2014). Phospholipase A2 Isolated from the Venom of Crotalus durissus terrificus Inactivates Dengue virus and Other Enveloped Viruses by Disrupting the Viral Envelope. PLoS One.

[13] Harlow E, Lane DP (1988). Andibodies: A Laboratory Manual.

[14] Erhardt W, Hebestedt A, Aschenbrenner G, Pichotka B, Blümel G (1984). A comparative study with various anesthetics in mice (pentobarbitone, ketamine-xylazine, carfentanyl-etomidate). Res Exp Med (Berl).

[15] Dos Santos HW, Poloni TR, Souza KP, Muller VD, Tremeschin F, Nali LC, Fantinatti LR, Amarilla AA, Castro HL, Nunes MR (2008). A simple one-step real-time RT-PCR for diagnosis of dengue virus infection. J Med Virol.

[16] Chen HC, Hofman FM, Kung JT, Lin YD, Wu-Hsieh BA (2007). Both virus and tumor necrosis factor alpha are critical for endothelium damage in a mouse model of dengue virus-induced hemorrhage. J Virol.

[17] Yen YT, Chen HC, Lin YD, Shieh CC, Wu-Hsieh BA (2008). Enhancement by tumor necrosis factor alpha of dengue virus-induced endothelial cell production of reactive nitrogen and oxygen species is key to hemorrhage development. J Virol.

[18] Guabiraba R, Besnard AG, Marques RE, Maillet I, Fagundes CT, Conceição TM, Rust NM, Charreau S, Paris I, Lecron JC (2013). IL-22 modulates IL-17A production and controls inflammation and tissue damage in experimental dengue infection. Eur J Immunol.

[19] Guabiraba R, Marques RE, Besnard AG, Fagundes CT, Souza DG, Ryffel B, Teixeira MM (2010). Role of the chemokine receptors CCR1, CCR2 and CCR4 in the pathogenesis of experimental dengue infection in mice. PLoS One.

[20] Gonçalves D, De Queiroz Prado R, Almeida Xavier E, Cristina De Oliveira N, Da Matta Guedes PM, Da Silva JS, Moraes Figueiredo LT, Aquino VH (2012). Imunocompetent mice model for dengue virus infection. ScientificWorldJournal.

[21] Styer LM, Kent KA, Albright RG, Bennett CJ, Kramer LD, Bernard KA (2007). Mosquitoes inoculate high doses of West Nile virus as they probe and feed on live hosts. PLoS Pathog.

[22] Shresta S, Kyle JL, Snider HM, Basavapatna M, Beatty PR, Harris E (2004). Interferon-dependent immunity is essential for resistance to primary dengue virus infection in mice, whereas T- and B-cell-dependent immunity are less critical. J Virol.

[23] Halstead S (1970). Observations related to pathogensis of dengue hemorrhagic fever. VI. Hypotheses and discussion. Yale J Biol Med.

[24] Dejnirattisai W, Jumnainsong A, Onsirisakul N, Fitton P, Vasanawathana S, Limpitikul W, Puttikhunt C, Edwards C, Duangchinda T, Supasa S (2010). Cross-reacting antibodies enhance dengue virus infection in humans. Science.

[25] Chareonsirisuthigul T, Kalayanarooj S, Ubol S (2007). Dengue virus (DENV) antibody-dependent enhancement of infection upregulates the production of anti-inflammatory cytokines, but suppresses anti-DENV free radical and pro-inflammatory cytokine production, in THP-1 cells. J Gen Virol.

[26] Ubol S, Phuklia W, Kalayanarooj S, Modhiran N (2010). Mechanisms of immune evasion induced by a complex of dengue virus and preexisting enhancing antibodies. J Infect Dis.

[27] Cardosa MJ (1987). Dengue virus isolation by antibody-dependent enhancement of infectivity in macrophages. Lancet.

[28] Jessie K, Fong M, Devi S, Lam S, Wong K (2004). Localization of dengue virus in naturally infected human tissues, by immunohistochemistry and in situ hybridization. J Infect Dis.

[29] Balsitis SJ, Coloma J, Castro G, Alava A, Flores D, McKerrow JH, Beatty PR, Harris E (2009). Tropism of dengue virus in mice and humans defined by viral nonstructural protein 3-specific immunostaining. Am J Trop Med Hyg.

[30] Kyle J, Beatty P, Harris E (2007). Dengue virus infects macrophages and dendritic cells in a mouse model of infection. J Infect Dis.

[31] Bhoopat L, Bhamarapravati N, Attasiri C, Yoksarn S, Chaiwun B, Khunamornpong S, Sirisanthana V (1996). Immunohistochemical characterization of a new monoclonal antibody reactive with dengue virus-infected cells in frozen tissue using immunoperoxidase technique. Asian Pac J Allergy Immunol.

[32] Ramos C, Sánchez G, Pando RH, Baquera J, Hernández D, Mota J, Ramos J, Flores A, Llausás E (1998). Dengue virus in the brain of a fatal case of hemorrhagic dengue fever. J Neurovirol.

[33] Couvelard A, Marianneau P, Bedel C, Drouet MT, Vachon F, Hénin D, Deubel V (1999). Report of a fatal case of dengue infection with hepatitis: demonstration of dengue antigens in hepatocytes and liver apoptosis. Hum Pathol.

[34] Bente DA, Rico-Hesse R (2006). Models of dengue virus infection. Drug Discov Today Dis Models.

[35] Ferreira GP, Figueiredo LB, Coelho LF, PA S, Cecilio AB, Ferreira PC, Bonjardim CA, Arantes RM, Campos MA, Kroon EG (2010). Dengue virus 3 clinical isolates show different patterns of virulence in experimental mice infection. Microbes Infect.

[36] Rico-Hesse R (2010). Dengue virus virulence and transmission determinants. Curr Top Microbiol Immunol.

[37] Armstrong PM, Rico-Hesse R (2001). Differential susceptibility of Aedes aegypti to infection by the American and Southeast Asian genotypes of dengue type 2 virus. Vector Borne Zoonotic Dis.

[38] Libraty D, Young P, Pickering D, Endy T, Kalayanarooj S, Green S, Vaughn D, Nisalak A, Ennis F, Rothman A (2002). High circulating levels of the dengue virus nonstructural protein NS1 early in dengue illness correlate with the development of dengue hemorrhagic fever. J Infect Dis.

[39] Vaughn D, Green S, Kalayanarooj S, Innis B, Nimmannitya S, Suntayakorn S, Endy T, Raengsakulrach B, Rothman A, Ennis F (2000). Dengue viremia titer, antibody response pattern, and virus serotype correlate with disease severity. J Infect Dis.

[40] Silveira GF, Meyer F, Delfraro A, Mosimann AL, Coluchi N, Vasquez C, Probst CM, Báfica A, Bordignon J, Dos Santos CN (2011). Dengue virus type 3 isolated from a fatal case with visceral complications induces enhanced proinflammatory responses and apoptosis of human dendritic cells. J Virol.

[41] Watts DM, Porter KR, Putvatana P, Vasquez B, Calampa C, Hayes CG, Halstead SB (1999). Failure of secondary infection with American genotype dengue 2 to cause dengue haemorrhagic fever. Lancet.

[42] Kanakaratne N, Wahala WM, Messer WB, Tissera HA, Shahani A, Abeysinghe N, De-Silva AM, Gunasekera M (2009). Severe dengue epidemics in Sri Lanka, 2003–2006. Emerg Infect Dis.

[43] Alfonso HL, Amarilla AA, Goncalves PF, Barros MT, de Almeida FT, Silva TR, et al. Phylogenetic relationship of dengue virus type 3 isolated in Brazil and Paraguay and global evolutionary divergence dynamics. Virol J. 2012;9. 10.1186/1743-422X-9-124 PMC3494512 22716071

[44] Messer W, Gubler D, Harris E, Sivananthan K, de Silva A (2003). Emergence and global spread of a dengue serotype 3, subtype III virus. Emerg Infect Dis.

[45] SVS SdVeS: Relatório de casos de dengue–2008. In*.*; 2009.

[46] Ruoslahti E (1996). RGD and other recognition sequences for integrins. Annu Rev Cell Dev Biol.

[47] Pancharoen C, Thisyakorn U (2001). Neurological manifestations in dengue patients. Southeast Asian J Trop Med Public Health.

[48] Puccioni-Sohler M, Orsini M, Soares CN (2012). Dengue: a new challenge for neurology. Neurol Int.

[49] Miagostovich MP, Ramos RG, Nicol AF, Nogueira RM, Cuzzi-Maya T, Oliveira AV, Marchevsky RS, Mesquita RP, Schatzmayr HG (1997). Retrospective study on dengue fatal cases. Clin Neuropathol.

[50] Nogueira RM, Filippis AM, Coelho JM, Sequeira PC, Schatzmayr HG, Paiva FG, Ramos AM, Miagostovich MP (2002). Dengue virus infection of the central nervous system (CNS): a case report from Brazil. Southeast Asian J Trop Med Public Health.

[51] Solomon T, Dung NM, Vaughn DW, Kneen R, Thao LT, Raengsakulrach B, Loan HT, Day NP, Farrar J, Myint KS (2000). Neurological manifestations of dengue infection. Lancet.

[52] Liou ML, Hsu CY (1998). Japanese encephalitis virus is transported across the cerebral blood vessels by endocytosis in mouse brain. Cell Tissue Res.

[53] Kobiler D, Lustig S, Gozes Y, Ben-Nathan D, Akov Y (1989). Sodium dodecylsulphate induces a breach in the blood–brain barrier and enables a West Nile virus variant to penetrate into mouse brain. Brain Res.

[54] Lustig S, Danenberg HD, Kafri Y, Kobiler D, Ben-Nathan D (1992). Viral neuroinvasion and encephalitis induced by lipopolysaccharide and its mediators. J Exp Med.

[55] McGavern DB, Kang SS (2011). Illuminating viral infections in the nervous system. Nat Rev Immunol.

[56] Koyuncu OO, Hogue IB, Enquist LW (2013). Virus infections in the nervous system. Cell Host Microbe.

[57] Mathur A, Khanna N, Chaturvedi UC (1992). Breakdown of blood–brain barrier by virus-induced cytokine during Japanese encephalitis virus infection. Int J Exp Pathol.

[58] Kobayashi Z, Tsuchiya K, Yokota O, Haga C, Arai T, Akiyama H, Kotera M, Mizusawa H (2009). Japanese encephalitis - serial CT findings and neuropathology in an autopsy case. Clin Neuropathol.

[59] Rodriguez-Madoz JR, Bernal-Rubio D, Kaminski D, Boyd K, Fernandez-Sesma A (2010). Dengue virus inhibits the production of type I interferon in primary human dendritic cells. J Virol.

[60] Halstead S, O’Rourke E (1977). Dengue viruses and mononuclear phagocytes. I. Infection enhancement by non-neutralizing antibody. J Exp Med.

[61] Zellweger RM, Prestwood TR, Shresta S (2010). Enhanced infection of liver sinusoidal endothelial cells in a mouse model of antibody-induced severe dengue disease. Cell Host Microbe.

